# Quarantine and National Board of Health

**Published:** 1880-06

**Authors:** Jerome Cochran, J. B. Gaston, S. D. Seeley, J. S. Weatherly, M. H. Jordan, Geo. A. Ketchum, C. D. Parke, P. Bryce, J. J. Dement, Geo. E. Kumpe

**Affiliations:** Committee of Public Health; Committee of Public Health; Committee of Public Health; Committee of Public Health; Committee of Public Health; Committee of Public Health; Committee of Public Health; Committee of Public Health; Committee of Public Health; Committee of Public Health


### QUARANTINE AND NATIONAL BOARD OF HEALTH.

Since the signal failure in 1879 to prevent or arrest yellow fever at Memphis, by quarantine, the theory of prevention upon which the Natlonal Board ot Health was organized, has not been so generally advocated. The proof, in that case, was conclusive against protection by quarantine. A few journals, however, still insist that the Board is a necessity to prevent importation and transportation of the disease, notwithstanding the protestations of those sought to be protected. The "Sanitarian" comes forward with the most unreasonable and inconsistent arguments in defense of the Board and in favor of the proposed increase of its power.

The Sanitarian for May says: "But attention was early diverted from it (inspection of seaport quarantines) by the outbreak of yellow fever at Memphis, and the efforts of the Board for its limitation and suppression were exercised with a degree of skill rarely if ever equaled, and surely never surpassed, in any age or country in battling with an epidemic, or, all things considered, crowned with more success." Of New Orleans, the same journal says: "* * By joint action of the National Board and the local health authorities, every case was made the centre of special and extended sanitation, so complete as to literally stamp out the disease."

We do not remember ever to have read in the same article more inconsistent and contradictory statements than the above. In New Orleans the disease was "literally stamped out" by the Board and local health authorities, and yet, in Memphis, "the efforts of the Board for its limitation and suppression" were never "crowned with more signal success." We all know that the disease extended and continued without abatement for months in Memphis, and this is called successful efforts of the Board for its limitation and suppression. The truth of the whole matter was made plain last year, and will be understood notwithstanding the mystification, by strong expressions, attempted by the Sanitarian. The local cause of fever existed in Memphis, and the disease was contracted and prevailed to a destructive extent, notwithstanding the strict quarantine and stamping out operations of the Board. In New Orleans the condition was fortunately different. The cause did not exist to any extent and the fever did not prevail. The apologists for the Board give it glory alike when the disease does prevail and when it does not. They are bent on giving protection by quarantine because they are on that line.

Those immediately affected by the infamous nuisance, have great feeling on the subject, and send up memorials to Congress against extending the powers of the Board. In our last issue we published the protest of the Savannah people on this subject, and give below a memorial from Alabama:

THE MEMORIAL OF THE BOARD OF HEALTH OF THE STATE OF ALABAMA.

*To the Honorable the Senate and the House of Representatives of the Congress of the United States of America:*

The Board of Health of the State of Alabama, having duly considered the scope and tendency of the several bills now pending in the Congress of the United States "To increase the efficiency of the National Board of Health," have reached, in reference to the same, the following general conclusions, namely:

(1.) That it is the duty as well as the privilege of every community, as well as of every individual, to take care of itself to the full extent of its ability to do so, and, amongst other things, to protect itself against the invasion of epidemic diseases.

(2.) That the practical questions involved in the establishment and management of quarantines, are of such grave importance, and affect so intimately and so profoundly the material interests of the State, and of the local communities within the State, as to make it neither wise nor prudent for us to intrust the administration of quarantine to the hands of any other health authorities than those who are of our own appointment and directly responsible to our own people.

(3.) That while the several bills now pending in the national congress "To increase the efficiency of the National Board of Health," do not interfere with the right of the State and local authorities to proclaim and enforce such quarantine regulations, in addition to those of the National Board, as to them may seem advisable, they are still open to objection from the fact that they give to the National Board the power to establish and administer quarantines within the limits of the States against all commerce and travel of which one of the terminal points lies outside of the State, and this without the consent of the local authorities, and even without consultation with them.

(4.) That it seems to us to be a proposition clearly self-evident to all persons of competent judgment in questions of this kind, that the State can not afford to allow this large grant of power, so nearly affecting the welfare of our people, to be placed in the hands of the National Board of Health, or of any other agent of the federal government, without making earnest efforts to prevent it.

(5.) That without entering into the discussion of the principles and circumstances under which it may become advisable for the general government to extend to State and municipal authorities pecuniary and other assistance towards the establishment and maintenance of quarantines, it still seems perfectly clear to us that the proclamation and administration of quarantines should, in all cases, be reserved to the State and municipal authorities.

(6.) That it also seems to us to be true, beyond all reasonable question, that no uniform system of quarantine regulations, suitable to all times and places, can possibly be devised; but, *per contra*, that the quarantine regulations that are applicable to one place will often prove entirely unsuited to the wants of another place; and that in different seasons even the same place will require widely different regulations.

(7.) That it is the circumstantial details of quarantines that present the practically difficult and important problems of quarantine administration; and that these can not be wisely ordered nor wisely managed except by experts, who are intimately acquainted with local and surrounding conditions.

(8.) That these propositions being admitted, it follows that the rule established last year by the National Board of Health, to the effect that assistance sheuld be extended only to such State and municipal boards as had first adopted the national rules and regulations, is very gravely objectionable, both in principle and practice, and ought not to be continued.

(9.) That the only wise and expedient rule in this regard is this, namely: That such State and municipal boards as desire the assistance of the National Board, should be required to submit their own local regulations to the National Board of examination, and that if these are found by the National Board to be of reasonable thoroughness and sufficiency, the needed assistance should then be granted.

(10.) That the National Board ought, properly, to have the general direction and control of quarantines against foreign countries; but that even these international quarantines could be most wisely and efficiently administered through the agency of the State and municipal boards having local jurisdiction over our seaport cities.

(11. That we are in no sense antagonistic to the National Board; but, contrarywise, appreciate very fully that it has for the exercise of its legitimate functions a wide and important field of usefulness, within which the State and municipal boards have no jurisdiction, and this without emasculating and weakening the State and municipal boards, and without absorbing, directly or indirectly, their most important functions.

(12.) That holding these opinions, we should very much regret to see the power and usefulness of the National Board, in its own proper field of action, diminished or destroyed by the withholding of the appropriations for which it has made application, and which are indubitably necessary for the successful continuance of the scientific investigations, sanitary surveys, and other works of sanitary administration and research which it has auspiciously begun.

All of which is respectfully submitted, in the name and behalf of the Board of Health of the State of Alabama.

Done in the City of Montgomery, on the 10th day of May, *Anno Domini*, 1880.

Jerome Cochran, M.D.,
J. B. Gaston, M.D.,
S. D. Seeley, M.D.,
J. S. Weatherly, M.D.,
M. H. Jordan, M.D.,
Geo. A. Ketchum, M.D.,
C. D. Parke, M.D.,
P. Bryce, M.D.,
J. J. Dement, M.D.,
Geo. E. Kumpe, M.D.,
*Committee of Public Hea'th.*

